# An insulin-like growth factor-like peptide promotes ovarian development in the silkmoth *Bombyx mori*

**DOI:** 10.1038/s41598-019-54962-w

**Published:** 2019-12-05

**Authors:** Daiki Fujinaga, Kunihiro Shiomi, Yoshimasa Yagi, Hiroshi Kataoka, Akira Mizoguchi

**Affiliations:** 1 Department of Integrated Biosciences, Graduate School of Frontier Sciences, The University of Tokyo, Kashiwa, Chiba, 277-8562 Japan; 2 Faculty of Textile Science and Technology, Shinshu University, Ueda, 386-8567 Japan; 3 Division of Biological Science, Graduate School of Science, Nagoya University, Nagoya, 464-8602 Japan; 4 Division of Liberal Arts and Sciences, Aichi Gakuin University, Nisshin, Aichi 470-0195 Japan

**Keywords:** Biological metamorphosis, Entomology

## Abstract

Insulin family peptides are known to be key regulators of growth and metabolism in insects and vertebrates. Insects have two types of insulin family peptides: insulin-like peptides and insulin-like growth factor (IGF)-like peptides (IGFLPs). We recently demonstrated that an IGFLP in the silkmoth, *Bombyx mori* (BIGFLP) promotes the growth of the genital imaginal disc *ex vivo*. However, the role of BIGFLP in the regulation of insect growth remains unclear because no *in vivo* study has been performed. Therefore, we analysed the functions of BIGFLP *in vivo* by constructing *BIGFLP* knock-out (KO) *B*. *mori* using the clustered regularly interspaced palindromic repeats (CRISPR) and CRISPR-associated protein 9 (CRISPR-Cas9) system. The KO moths exhibited decreased body weights and size of the appendages compared wild-type (wt) moths. Interestingly, KO females also had drastically lower ovary weights and number of eggs than wt females. However, mutant ovaries that were transplanted into wt host pupae reached a similar weight to wt ovaries that were transplanted into the wt hosts, suggesting that IGFLP in the haemolymph promotes ovarian development. These findings show that BIGFLP regulates the growth and development of adult organs, particularly the ovaries, in *B*. *mori*.

## Introduction

Animal growth and development are regulated by a wide variety of hormones, among which insulin family peptides are particularly important. In vertebrates, an increase in blood glucose levels stimulates the secretion of insulin, which regulates sugar and lipid metabolism in the peripheral tissues^1,2^, while another class of insulin family peptides, insulin-like growth factors (IGFs), are known to promote whole-body growth and organ development^3^. The secretion of IGF-I is induced by other hormones, such as growth hormone and oestrogen, in mammals^4^.

Two types of insulin family peptides have been identified in insects: insulin-like peptides (ILPs) and IGF-like peptides (IGFLPs)^5^. Although these peptides have similar amino acid sequences, ILPs resemble insulin while IGFLPs resemble IGF in vertebrates in many ways^5^. An insect ILP was first discovered in the silkmoth *Bombyx mori* and named bombyxin^6^, followed by identification of approximately 40 bombyxin genes in the *B. mori *genome^7^ and of its homologues in the genomes of all insect species examined to date^8^. Many studies have shown that ILPs are involved in the regulation of metabolism, growth, longevity and stress responses^9,10^. ILPs are mainly expressed in the insulin-producing cells (IPCs) in the brain and are released into the haemolymph in response to feeding signals^9,10^, whereby they activate insulin/IGF signalling (IIS) in the peripheral tissues to promote cellular growth and metabolism^11^. Consequently, ILPs are regarded as key hormones that connect the nutritional status of an organism to tissue growth^5,12^.

An IGFLP was also first discovered in *B*. *mori*^13^. Subsequently, one of the *Drosophila* ILPs (Dilp6) was identified as a *Drosophila* counterpart of *Bombyx* IGFLP^14,15^. IGFLPs are mainly produced by the fat body during adult development with gene expression induced by the moulting hormone (ecdysteroids). However, *BIGFLP* is also expressed in the IPCs throughout development from the fourth instar onwards as well as in the testis sheaths and ovarioles during adult development^16^. Here, it has to be noted that the gene product of *BIGFLP* in the IPCs may not be an IGFLP but an ILP (a type of bombyxin) because the BIGFLP peptide has potential cleavage sites conserved in ILPs within its amino acid sequence and IPCs must have proprotein convertases as evidenced by the production of bombyxins. *Ex vivo* studies in *B*. *mori* have shown that purified BIGFLP promotes the growth of adult organs, such as the male genital disc, via the IIS pathway^13,17^. In *D*. *melanogaster*, it has been shown that *Dilp6* knock-out (KO) flies exhibited a decreased body mass and a smaller number of wing cells than wild-type (wt) flies, suggesting that Dilp6 promotes organ growth and systemic growth in flies^14,15^. Furthermore, the inhibition of *Dilp6* expression after pupation by RNA interference reduces the consumption of triacylglycerol and glycogen^15^. Thus, it appears that Dilp6 regulates the metabolism and growth of adult organs during development.

So far, *in vivo* analyses of the physiological functions of ILPs and IGFLPs have been performed mainly in *D*. *melanogaster* using genetic approaches, whereas functional analyses of these hormones in *B*. *mori* and other Lepidoptera have been limited to those *ex vivo* until very recently, due to the difficulties in applying genetic approaches. However, it is evident that both *in vivo* and *ex vivo* studies are necessary to establish the functions of hormones and to reveal molecular and cellular mechanisms of hormone actions. Furthermore, it is important to study a variety of insects to reveal general and species-specific features of hormone functions.

An innovative approach to analyse *B*. *mori* ILP (bombyxin) functions *in vivo* has recently been reported in which secretion of bombyxins from IPCs was inhibited by using an improved Gal4-UAS system and tetanus toxin that blocks synaptic transmission^18^. This study revealed that genetic silencing of the IPCs resulted in a decrease in the growth rate of *B*. *mori* larvae, confirming the involvement of ILPs in the regulation of larval growth.

In the present study, we investigated the functions of BIGFLP *in vivo* by constructing *BIGFLP* KO *B*. *mori* using the clustered regularly interspaced short palindromic repeat (CRISPR) and CRISPR-associated protein 9 (CRISPR-Cas9) system^19^. Phenotypic analyses of the CRISPR-Cas9-based *BIGFLP* KO mutants clearly showed that *BIGFLP* is responsible for organ growth and particularly development of the ovaries during pupa-adult development in *B*. *mori*.

## Results

### Genome editing

**Figure 1:**
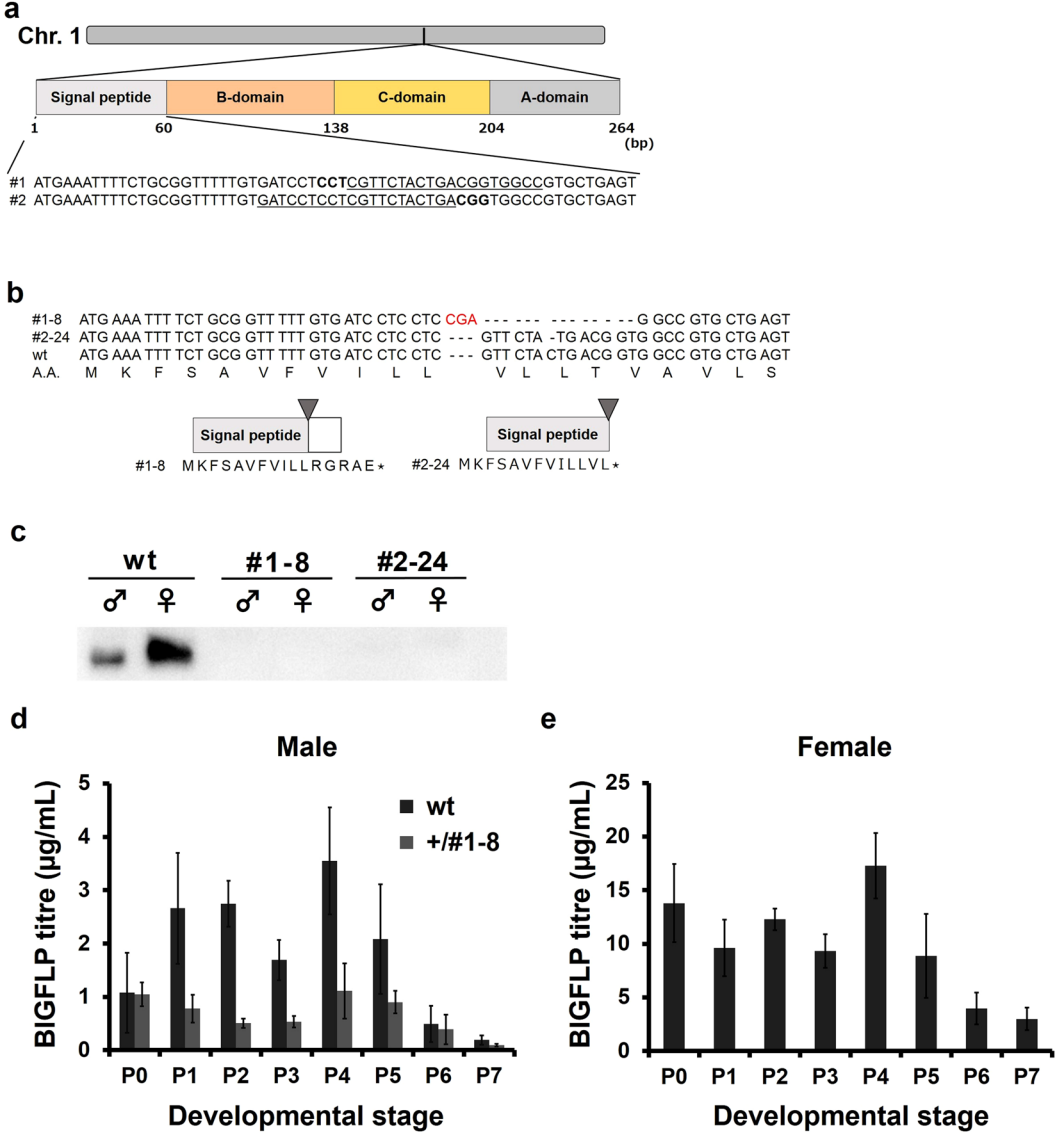
Construction of *BIGFLP* KO *B*. *mori* using the CRISPR-Cas9 system. (**a**) Schematic diagram of the *BIGFLP* locus and targeted DNA sequences. Two sgRNAs were designed to target the secretion signal peptide. (**b**) DNA sequences and corresponding amino acid sequences in the wt and two mutant strains (#1–8 and #2–24). Note that the translational frames of the DNA sequences in both mutants were shifted to produce a stop codon within the sequence that codes for the signal peptide. (**c**) Detection of BIGFLP signals in the P0 haemolymph by Western blotting. Full-length blots are presented in Supplementary Fig. S1. (**d**,**e**) Changes in the BIGFLP titres in the haemolymph during pupa-adult development in males (**d**) and females. (**e**) The titres were determined by TR-FIA. Values are means ± SD (n = 6). Pn: n days after pupation.

BIGFLP is encoded on chromosome 1 (the Z chromosome). Therefore, since *B*. *mori* is a female-heterogametic insect (ZZ in the male and ZW in the female), males have two *BIGFLP* loci and females have one. The precursor of BIGFLP consists of a secretory signal peptide and a B-domain, C-domain and A-domain^13^ (Fig. 1a). To induce mutation in the *BIGFLP* locus, CRISPR-Cas9 system-based genome editing was performed using the Kosetsu strain of *B*. *mori*. Two different sgRNAs were designed, both of which targeted the locus encoding the secretory signal peptide of preproBIGFLP (Fig. 1a). The synthesised sgRNA and Cas9 mRNA were injected into *B*. *mori* eggs and two mutant alleles were obtained in the next generation: one containing a 14-base deletion and 3-base insertion (#1–8) and one containing 1-base deletion (#2–24) (Fig. 1b). Both of these were considered to represent *BIGFLP* KO strains because of a frame-shift in the *BIGFLP* mRNA. BIGFLP was not detected in the pupal haemolymph of either strain (Fig. 1c), confirming that both were null mutants.

The BIGFLP titres in the haemolymph of wt males, heterozygous males (+/#1–8) and wt females were determined using time-resolved fluoroimmunoassay (TR-FIA) (Fig. 1d,e). (Note: No heterozygous mutant exists in females because *BIGFLP* is encoded on the Z chromosome.) The peak concentrations in wt males and females were similar to those observed for the Kinshu × Showa strain in our previous study^17^. However, two peaks were observed in wt individuals: at P1–P2 (1–2 days after pupation) and P4 in males and at P0 and P4 in females. The +/#1–8 males had lower titres than the wt males.

### Phenotypes of the KO strains

**Figure 2:**
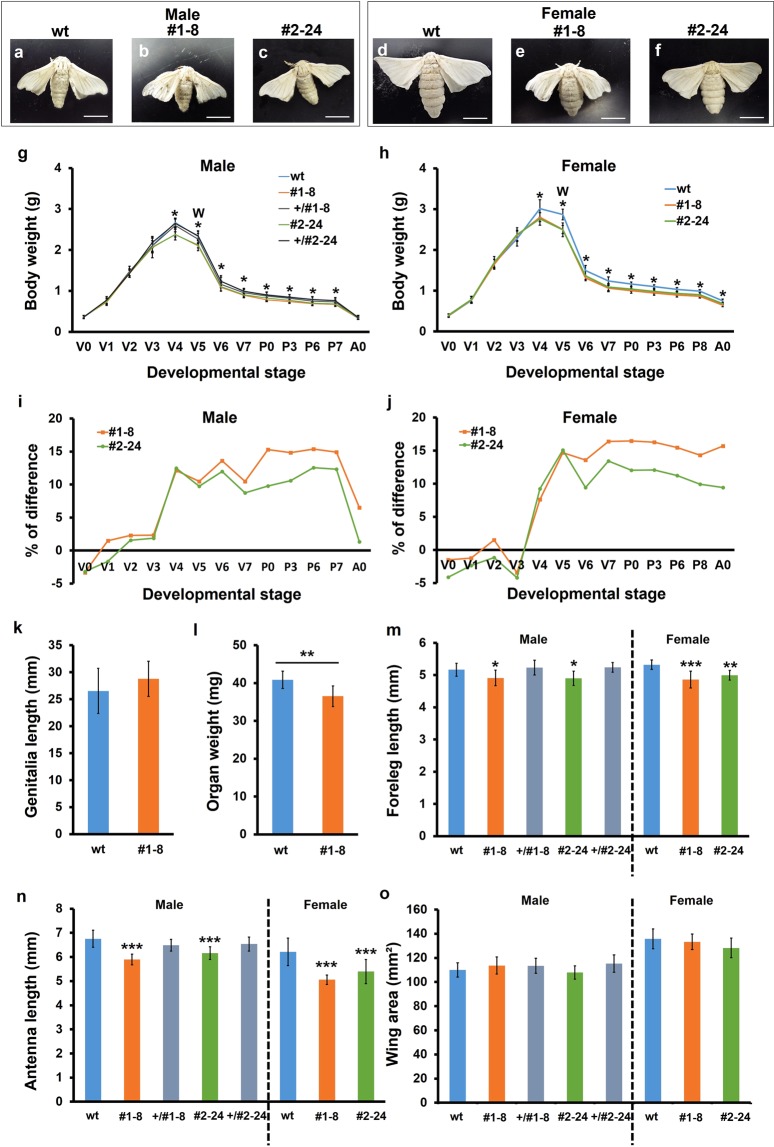
Phenotypic analyses of *BIGFLP* KO *B*. *mori*. (**a**–**f**) Representative images of adult moths. Scale bars: 1 cm. (**g**,**h**) Changes in the body weight during larva-pupa-adult development in males (**g**) and females (**h**). (**i**,**j**) The difference in body weight between the KO and wt moths. The percentage differences were calculated from the data shown in **g** and **h**. (**k**) Length of the genitalia of wt and KO males. (**l**) Weight of genitalia–testes complexes of wt and KO males. (**m**) Length of the adult forelegs (tibia and tarsus), (**n**) length of the antennae, and (**o**) area of the forewings of wt moths and heterozygous and homozygous mutants. Values are means ± SD (n = 17 in (**g**,**h**) n = 6 in (**k)** n = 7 in (**l**) n = 10 in (**m**–**o**). Asterisks in (**g**,**h**,**l–n**) indicate significant differences between wt and KO males and females (Student’s *t*-test (**k** and **l**) or Dunnett’s test (**g**,**h**,**m**–**o**) *p < 0.05; **p < 0.01; ***p < 0.001). Vn: n days after ecdysis to the fifth instar; W: day of wandering; Pn: n days after pupation; A0: day of adult emergence.

Adults of both KO strains were slightly smaller than wt adults, although no specific morphological differences were observed (Fig. 2a–f). For both sexes, the KO strains had significantly lower body weights than the wt strain from 1 day before the onset of wandering (Fig. 2g,h), suggesting that the *BIGFLP* gene product affects the growth of larvae towards the end of the feeding stage. No significant difference in body weight was detected between the KO and wt strains in adult males, whereas the difference was maintained in adult females (Fig. 2g–j). Neither of the KO strains exhibited any developmental delay or mortality.

Previous *ex vivo* analyses have demonstrated that BIGFLP promotes the growth of the genital imaginal discs^13,17^. Therefore, we first compared the length of the long axis and weight of the male genitalia in adults. Because male genitalia could not be isolated without any loss of content, the genitalia–testes complex was weighed instead. The length of the male genitalia was not different between wt and #1–8 males, while the weight of the genitalia–testes complex of #1–8 males was significantly lower than that of wt males (Fig. 2k,l), consistent with the previous results obtained *ex vivo*^17^.

We next investigated the effects of BIGFLP on the growth of other organs by measuring the size of the appendages (Fig. 2m–o). The forelegs (tibia and tarsus) and antennae were significantly shorter in both KO strains than in the wt strain (Fig. 2m,n). By contrast, no significant difference in the area of forewings was detected between the KO strains and wt strain (Fig. 2o). Heterozygous males did not show any significant decrease in the size of their organs. Together, these results demonstrate that the *BIGFLP* gene product is responsible for the growth of adult organs, although its effect varies among different organs.

### Gonadal development

**Figure 3:**
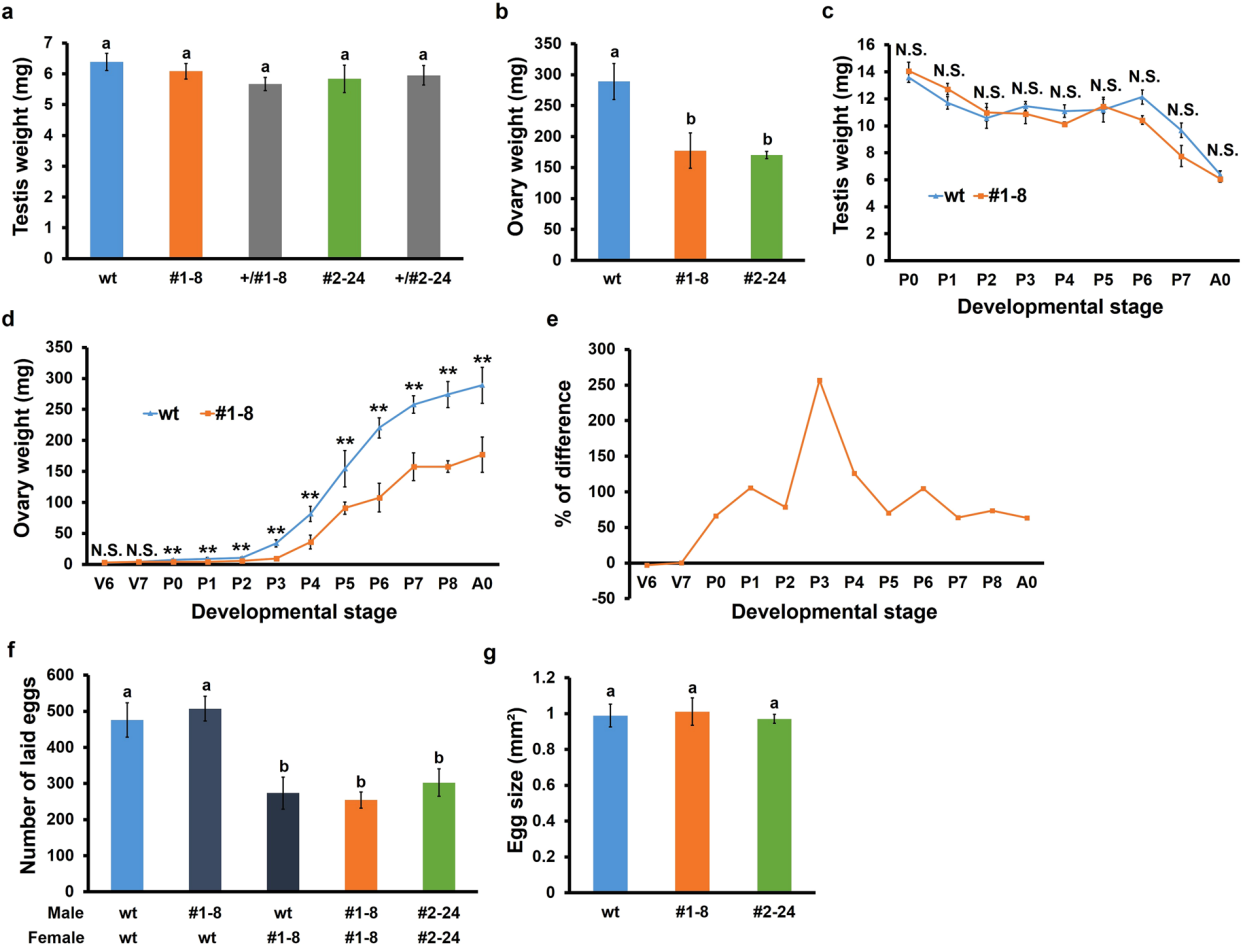
Effects of *BIGFLP* KO on gonadal development in *B*. *mori*. (**a**,**b**) Weight of the testes (**a**) and ovaries (**b**) of day-0 adults. (**c**,**d**) Changes in the weight of the testes from P0 to A0 (**c**) and ovaries from V6 to A0 (**d**). (**e**) The difference in ovary weight between the KO and wt females. (**f**) Numbers of eggs laid within 24 h of mating–the combinations of male and female genotypes are indicated below the graph. (**g**) Sizes of the laid eggs. Values are means ± SD (n = 6). Bars with different letters and asterisks indicate significant differences between groups [Tukey–Kramer’s test (**a**,**b**,**f**,**g**) or Student’s *t*-test (**c**,**d**) ^**^p < 0.01; N.S., not significant]. Vn: n days after ecdysis to the fifth instar; Pn: n days after pupation; A0: the day of adult emergence.

There was a significant difference in the body weight of adult females but not adult males between the wt and KO strains, suggesting that a difference in the weight of some sex-specific tissues may contribute to the observed difference in body weight. Therefore, the weight of the testes and ovaries in KO and wt adults was compared (Fig. 3a,b). There was no significant difference in the weight of the testes between KO and wt males, whereas the weight of the ovaries was remarkably lower in KO females than in wt females (39% decrease in #1–8; 42% decrease in #2–24).

To determine the critical time at which *BIGFLP* affects gonadal development, the testes and ovaries of the wt and #1–8 strains were weighed daily throughout post-wandering stages (Fig. 3c,d). No significant difference in testis weight was observed between the two strains at any time (Fig. 3c). However, the ovaries of the KO strain had a lower weight at as early as P0, following which the difference gradually increased to reach a plateau at around P6 (Fig. 3d,e). This observation suggests that the *BIGFLP* gene product promotes ovarian development during the first half of adult development.

To identify the cause of the observed decrease in the ovary weight, the number and size of laid eggs were examined. It was found that females of both KO strains laid significantly fewer eggs than wt females (Fig. 3f). The genotypes of the mated males did not affect the number of eggs, suggesting that *BIGFLP* affects neither the mating behaviour nor reproductive potential of males. Furthermore, there was no difference in the size of the laid eggs between the KO and wt strains (Fig. 3g) and most eggs developed normally even in the KO strains. Together, these results suggest that the *BIGFLP* gene product increases the number of female gametes but does not affect the fertilisation of these.

### Decrement of IIS in the mutant ovaries

**Figure 4:**
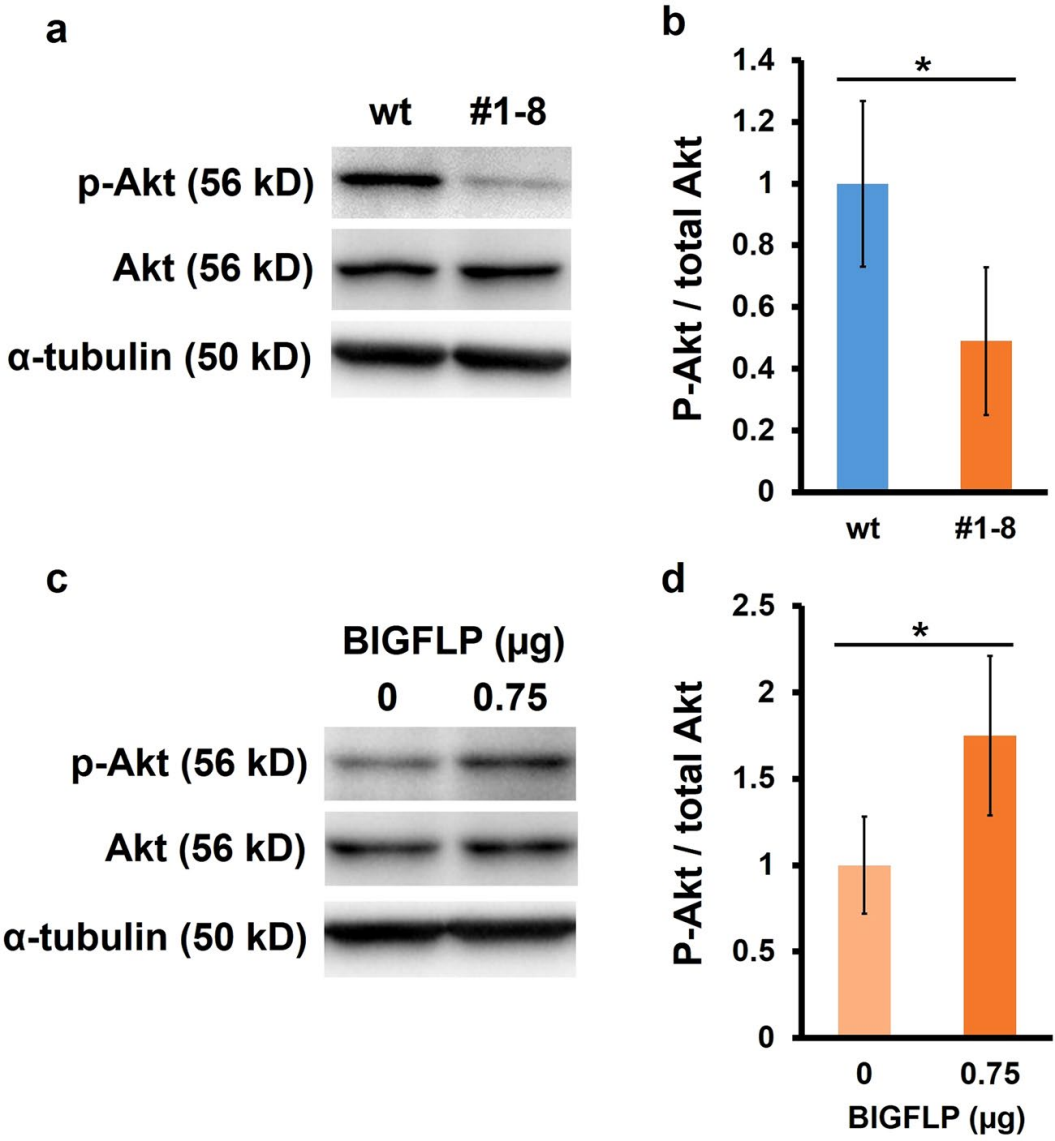
Intensity of IIS in the mutant ovaries of *B*. *mori* (**a**) Detection of Phosphorylated Akt (p-Akt), total Akt (Akt) and α-tubulin in the ovaries of day-0 pupae by Western blotting. Each set of blots (wt vs. #1–8) was cropped from the same blot. Full-length blots are presented in Supplementary Fig. S2. (**b**) Signal intensity of p-Akt normalised to Akt. (**c**) Detection of p-Akt, total Akt and α-tubulin by Western blotting 30 min after the injection of BIGFLP into #1–8 P0 females. Each set of blots (0 µg vs. 0.75 µg) was cropped from the blot. Full-length blots are presented in Supplementary Fig. S3. (**d**) Signal intensity of p-Akt normalised to Akt 30 min after the injection of BIGFLP. Values are means ± SD (n = 4). Asterisks indicate significant differences between groups (Student’s *t*-test: *p < 0.05). Akt was detected after the incubation of ovary extract with alkaline phosphatase.

We recently reported that BIGFLP promotes the growth of the male genital disc *ex vivo* via the IIS pathway^17^. To investigate the mechanisms of BIGFLP action on the ovary *in vivo*, we examined the phosphorylation status of one of the components of the IIS pathway, Akt, in the ovaries of P0 females by Western blotting. The signal intensity of phosphorylated Akt was significantly lower in the #1–8 strain compared with the wt strain (Fig. 4a,b). To confirm that IIS is stimulated by circulating BIGFLP, we injected purified BIGFLP (0.75 µg) into #1–8 pupae, which caused the signal intensity of phosphorylated Akt in the ovaries to significantly increase (Fig. 4c,d). Thus, it appears that BIGFLP activates the IIS pathway in the ovaries.

### Timing of BIGFLP action

**Figure 5:**
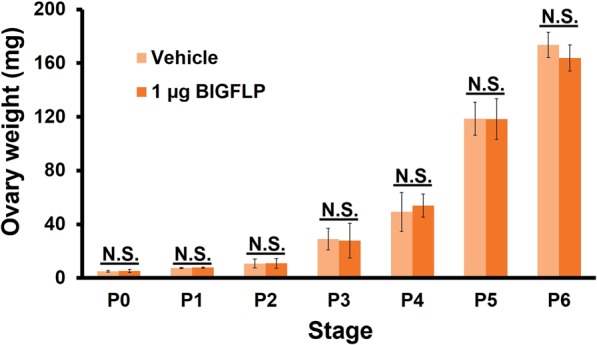
Effects of BIGFLP injection into KO *B*. *mori* pupae. BIGFLP (1 µg) or vehicle was injected into day-0 to day-6 KO (#1–8) female pupae and the weights of the ovaries were measured 24 h after injection. Values are means ± SD (n = 6). There were no significant differences between groups (Student’s *t*-test: N. S., not significant). Pn: n days after pupation.

To determine when BIGFLP promotes ovarian development, we injected BIGFLP into KO females at various times after pupation and measured the weight of the ovaries 24 h after the injection (Fig. 5). Contrary to our expectation, the administration of BIGFLP did not increase the weight of the ovaries at any stages. Therefore, we were unsuccessful in rescuing the decrease in ovary weight in KO females by BIGFLP injection. We considered that the injected BIGFLP was rapidly degraded in the haemolymph, preventing it from affecting the ovaries.

### Rescue of restricted ovarian development by ovary transplantation

To overcome the difficulty in rescuing a restricted development of the ovaries by BIGFLP injection, we next performed an ovary transplantation experiment in which one of the pair of ovaries of KO (mutant) females were exchanged with those of wt females at P0, because this treatment should enable the mutant ovaries to be continuously exposed to the *BIGFLP* gene product *in vivo*. As a control, ovaries were also exchanged between the same strains of pupae. This experiment also had another purpose, which is to determine the source of the *BIGFLP* gene product affecting ovarian development. A large amount of BIGFLP is present in the haemolymph of wt pupae (Fig. 1e). However, *BIGFLP* is also expressed in the testis sheaths and ovarioles during adult development^16^, raising the possibility that it is the BIGFLP that is produced in the gonad that promotes gonadal development in a paracrine manner. Therefore, it was necessary to test this possibility. We measured the weight of the transplanted ovaries after eclosion, predicting that if the circulating *BIGFLP* gene product is responsible for ovarian development, the implanted mutant ovary would gain a greater weight in the wt host, whereas if the BIGFLP produced by the ovary itself is responsible, development of the implanted mutant ovary would not be promoted in the wt host.

**Figure 6:**
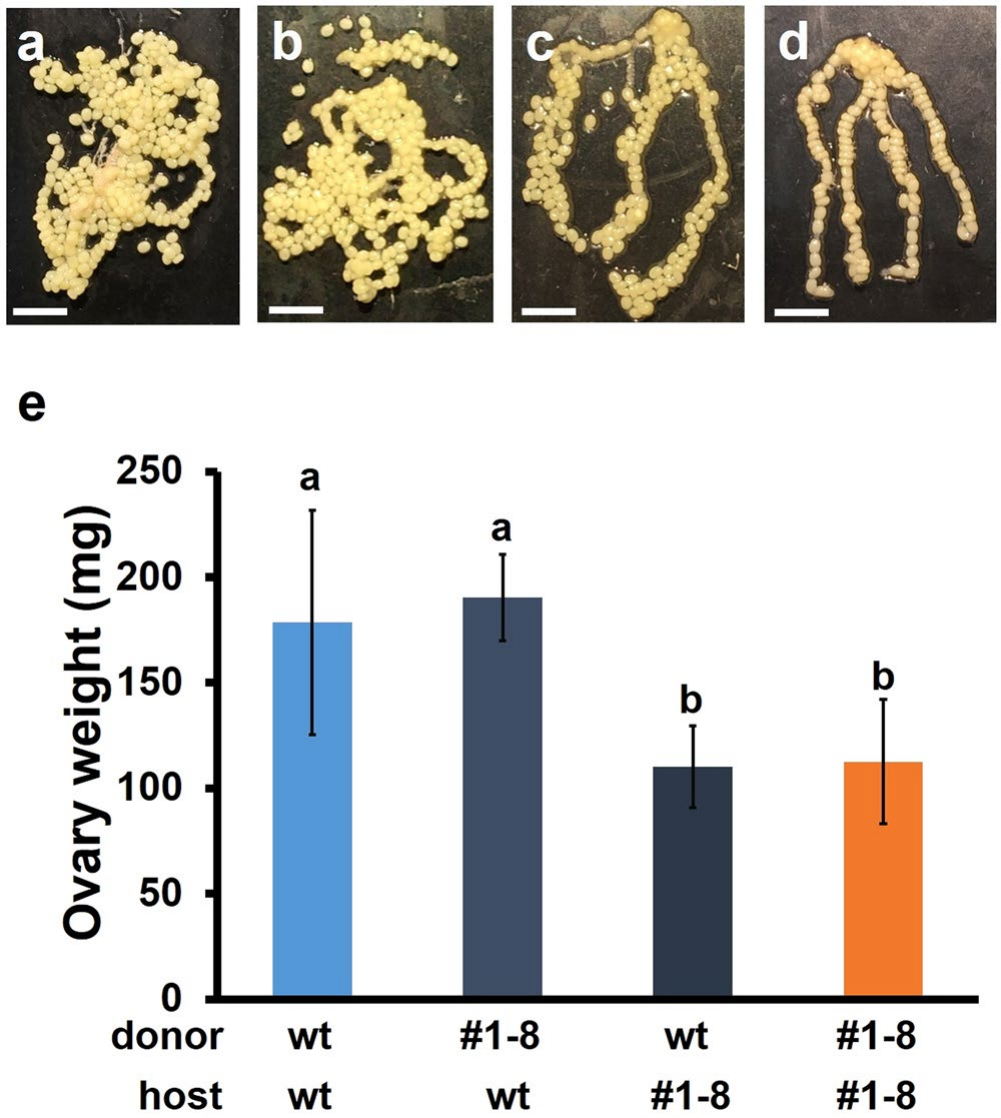
Effect of ovary transplantation between KO and wt *B*. *mori* pupae. A single ovary was dissected from KO and wt females within 6 h of pupation and transplanted into another individual. The implanted ovaries were then dissected after eclosion. (**a**–**d**) Representative images of a wt ovary transplanted into a wt host (**a**), a #1–8 ovary transplanted into a wt host (**b**), a wt ovary transplanted into a #1–8 host (**c**), and a #1–8 ovary transplanted into a #1-8 host (**d**). Scale bars: 5 mm. (**e**) Weights of wt or #1–8 ovaries (donor) transplanted into the same or different types of pupae (hosts). Values are means ± SD (n = 8–10). Bars with different letters are significantly different (Tukey–Kramer’s test: p < 0.05).

We found that the mutant ovaries that were transplanted into the wt hosts reached a similar weight to the wt ovaries that were transplanted into the wt hosts. By contrast, the wt ovaries that were transplanted into the mutant hosts were much smaller than the wt ovaries that were transplanted into the wt hosts and were comparable in weight to the mutant ovaries that were transplanted into the mutant hosts (Fig. 6a–e). The sizes of the eggs in the implanted ovaries were similar in all donor and host combinations (Fig. 6a–d). These results indicate that *BIGFLP* is undoubtedly responsible for ovarian development, and it is the *BIGFLP* gene product in the haemolymph that promotes ovarian development after pupation, with the BIGFLP that is produced by the ovary itself having little, if any, effect.

### Effect of brain removal in *BIGFLP* KO strains

**Figure 7:**
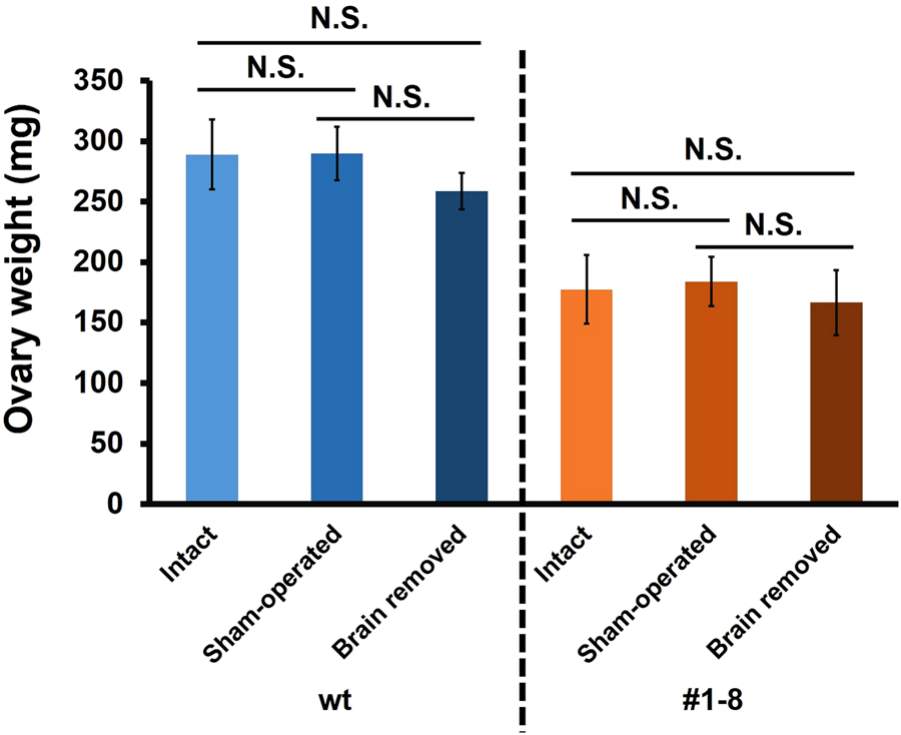
Effects of brain removal on ovarian development. The brain was removed from KO and wt females within 6 h of pupation, and the ovaries were weighed after eclosion. Values are means ± SD (n = 6). There were no significant differences between groups (Tukey–Kramer’s test: N. S., not significant).

The ovary transplantation experiment has shown that the gene product of *BIGFLP* in the haemolymph promotes ovarian development. However, it is unclear whether BIGFLP is the only factor regulating ovarian development because *BIGFLP* is also expressed in the brain, and its product may be an ILP but not an IGFLP. To address this question, we removed the brain at P0 from both wt and #1–8 females and weighted their ovaries after eclosion (Fig. 7). Although the ovary weights of brain-removed females seemed slightly lower than those of intact or sham-operated females, no significant differences in the weights were observed between the intact and brain-removed animals in both wt and #1–8 strains. These results indicate not only that the fat body-derived IGFLP is the major factor regulating ovarian development but that ILPs produced by the IPCs in the brain, including the gene product of *BIGFLP*, have little, if any, effect on ovarian development.

## Discussion

The present analysis of CRISPR-Cas9-based gene KO *B*. *mori* clearly showed that the *BIGFLP* gene product regulates the growth and development of the adult organs. The size of appendages such as the antennae and legs was significantly lower in BIGFLP-deficient mutants, suggesting that the *BIGFLP* gene product plays a role in promoting the growth of these organs. However, the most remarkable effect of *BIGFLP* KO was observed in the ovaries during pupa-adult development, with the weight of the ovaries and the number of eggs laid being reduced by approximately half in the KO mutants but the transplantation of mutant ovaries into wt pupae that expressed *BIGFLP* resulting in a nearly 50% increase in ovary weight. The decerebration experiments demonstrated that the main *BIGFLP* gene product involved in the regulation of ovarian development is the BIGFLP produced by the fat body.

In some insects, including *B*. *mori* and *D*. *melanogaster*, brain-derived ILPs are released in response to a feeding signal and activate the IIS pathway in the peripheral tissues to promote their growth during the feeding stage^9,10^. However, no feeding signal is available during pupa-adult development when the adult organs exhibit drastic growth and development. Therefore, another hormonal signal is likely required to activate the IIS pathway and promote tissue growth during this stage of insect development, and it is conceivable that IGFLP plays such a role.

The CRISPR-Cas9 system is a convenient method for constructing gene KO organisms. In the present study, two KO strains with two different sgRNAs were independently constructed (Fig. 1a) and three generations of backcrossing were performed to remove any off-target mutations. In addition, there were no differences in any of phenotypic characteristics between the #1–8 and #2–24 strains, indicating that the decreases in body weight and organ size that were observed in the KO strains did not result from off-target effects of the genome editing but rather were a direct result of the BIGFLP defect.

The KO larvae had a lower body weight than the wt larvae on day 4 of the fifth instar (Fig. 2g–j), which is a long time before BIGFLP is expressed in the fat body. It has previously been reported that *BIGFLP* is also expressed in the IPCs in the brains of fourth and fifth instar larvae^16^. The BIGFLP that is produced by the fat body is released as a single-chain peptide, like IGFs, despite the existence of potential cleavage sites in its molecule at the conserved positions that have been identified in various ILPs^13^, presumably because the fat body cells lack processing enzymes for cleaving this peptide. By contrast, the *BIGFLP* gene product in the IPCs is expected to be processed to generate a two-chain insulin-like structure in these cells. Thus, the IPC-derived *BIGFLP* gene product may not be an IGFLP but rather an ILP and may be released into the haemolymph together with other ILPs (bombyxins) from the IPCs and function as a member of the bombyxins. It has recently been reported that genetic blockade of bombyxin neural transmission using a Gal4-UAS system in *B*. *mori* led to a decrease in the growth rate of fifth instar larvae, demonstrating a role of bombyxins in the regulation of larval growth^18^. Therefore, it is possible that the observed difference in body weight between the KO and wt larvae at the mid-fifth instar (Fig. 2g–j) reflects the total amount of bombyxins in the haemolymph.

This then raises the question why the body weight decline in the KO strain is only manifested after several days in the fifth instar despite the *BIGFLP* gene being constantly expressed in this instar. It is assumed that the relative importance of this peptide among the bombyxins increases at the mid-fifth instar stage due to a decrease in the expression levels of other bombyxin genes or a decrease in the total bombyxin concentration in the haemolymph caused by the dramatic increase in haemolymph volume that is associated with rapid larval growth in the fifth instar.

After the onset of wandering, there was no change in the difference in body weight between the KO and wt strains in either sexes until eclosion (Fig. 2i,j). This is reasonable because gut-purged larvae and pupae do not eat or excrete anything. However, the difference in males disappeared after eclosion. Soon after eclosion, adult moths excrete the waste materials that accumulated during pupa-adult development in the form of meconium. Therefore, the observed disappearance of the difference in body weight between KO and wt males after eclosion suggests the possibility that wt animals had a larger volume of meconium. This, in turn, might reflect a higher metabolic activity of wt animals and thus indicate that BIGFLP enhances the metabolic activity of the pupae.

Satake *et al*.^20^ previously reported that the injection of bombyxin into the isolated abdomens of *B*. *mori* larvae led to a decrease in the contents of trehalose in the haemolymph and glycogen in the fat body, both of which are major storage carbohydrates in insects, and it has also recently been suggested that these carbohydrates are used for energy production^21^. Since only a single insulin/IGF-like receptor gene exists in the *B*. *mori* genome^8^, it is considered that bombyxins and BIGFLP act through the same receptor, with their functions compensating each other. Therefore, BIGFLP might support the growth of adult organs by activating energy metabolism. To test this hypothesis, the amounts of unused energy and material stocks for adult organ development must be compared between KO and wt males in the future. In females, the differences in the body weight between the strains did not disappear. This is possibly due to a large reduction in the ovary weights of the KO females.

In *D*. *melanogaster*, the downregulation of *Dilp6* expression after pupation results in a decrease in the consumption of triacylglycerol and glycogen^15^, suggesting that one of the roles of Dilp6 during metamorphosis is the activation of energy metabolism. Thus, the activation of energy metabolism may be a common action of insect IGFLPs during adult development, although these peptides may also have other functions.

A notable phenotype of the *Dilp6* mutant in *D*. *melanogaster* was the decrease in the body weights of post-feeding larvae, pupae and adults^14,15^. This phenotype is similar to that in the *BIGFLP* mutant of *B*. *mori* but is somewhat more severe, especially in adults: mutant adults had obviously lower body weights than wt even in males. This difference may be attributed to the difference in the number of *ILP/IGFLP* genes expressed during post-feeding stages between *B*. *mori* and *D*. *melanogaster*. In the genome of *D*. *melanogaster*, six *ILP/IGFLP* genes have been identified (*Dilp1*–*6*; note that *Dilp7* and *Dilp8* are considered relaxin-like genes)^10^, and only one of them, *Dilp6*, is strongly expressed during the pupal stage^14^. In contrast, *B*. *mori* has approximately 40 such genes in its genome, and three genes, *bombyxin-X1*, *-Y1* (identical to *BIGFLP*) and *-Z1*, are strongly expressed during the pupal stage in the fat body^7,22^. Therefore, it is conceivable that the gene products of *bombyxin-X1* and *-Z1* may also promote the growth of adult organs like BIGFLP does. The gene products of these genes are possibly IGFLPs rather than ILPs (bombyxins) because the fat body is not likely to possess proprotein convertases. It is unknown at present whether these peptides are actually released into the haemolymph. However, if the concentrations of these peptides are high enough to support the growth of adult organs, the presence of these IGFLPs in the haemolymph of *BIGFLP* KO pupae may explain the milder-than-expected phenotype of this mutant.

The KO moths had drastically lower ovary weights than the wt moths (Fig. 3d). Furthermore, although the KO moths had similar egg sizes to the wt moths, they laid approximately 50% fewer eggs (Fig. 3f,g), suggesting that the *BIGFLP* gene product increases the number of mature eggs. The mechanism by which *BIGFLP* regulates the number of mature eggs remains unclear but one possibility is that it is involved in the accumulation of energy and material stores for vitellogenesis. Since the body weight of wandering larvae was significantly lower in the *BIGFLP* KO strains (Fig. 2h), the mutant pupae may not have sufficient nutrients to support the production of a normal number of mature eggs.

However, since a large amount of BIGFLP is secreted only after pupal ecdysis, this peptide may exert its major effect(s) on ovarian development during pupa-adult development, although the effect of the brain-derived *BIGFLP* gene product on mid-fifth instar larvae may also contribute to egg production to some extent. The finding that the IIS signal level in the ovary as measured by Akt phosphorylation greatly increased in a BIGFLP-dependent manner (Fig. 4) indicates a possible mechanism by which BIGFLP regulates ovarian development. In *D*. *melanogaster*, the ovaries develop after eclosion and the number of eggs laid decreases under starving conditions^23–25^. Furthermore, it has been shown that the starvation-induced decrement of IIS in the ovaries induces programmed cell death of the nurse cells in the ovarian follicles^23^. In a eutrophic environment, the increased secretion of ILPs activates the IIS pathway in the ovaries, whereas under starving conditions, ILP secretion is inhibited and IIS decreases in the ovaries. These observations suggest that continuous activation of the IIS pathway in the ovary is necessary for normal follicle maturation. In the present study, BIGFLP injection into the KO pupae was unable to rescue the decrease in ovary weight (Fig. 5). However, mutant ovaries that had been transplanted into wt pupae attained a similar weight to control wt ovaries (Fig. 6), also suggesting that continuous activation of IIS is necessary for maintaining a normal number of follicles in the ovary. It is therefore possible that BIGFLP also activates the IIS pathway to prevent programmed cell death of the nurse cells in the ovaries.

Another possible mechanism by which BIGFLP regulates the number of eggs may be related to its effect on energy metabolism. As discussed above, this peptide may activate energy metabolism to support the growth of adult tissues. A substantial amount of energy is required to produce mature eggs and pupae do not incorporate any nutrition, meaning that the ovaries must develop using accumulated nutritional stocks. Therefore, it is conceivable that the KO female pupae were unable to efficiently use their stored nutritional resources to develop ovaries. To test this hypothesis, the amount of unused energy stores should be compared between KO and wt adult females in the future.

It has previously been demonstrated that ecdysteroids are involved in the regulation of ovarian development in *B*. *mori*^26^. For example, it was found that ovarian development did not occur when the abdomens of female *Bombyx* pupae were isolated from the anterior parts of the bodies by ligature immediately after pupal ecdysis but that the ovaries developed to reach an almost comparable size to normal mature ovaries when 20-hydroxyecdysone (20E) was injected into the isolated abdomens^27,28^, indicating that 20E is essential for inducing ovarian development. Therefore, we considered it possible that the reduced ovary weight in the KO females was caused by a decline in the ecdysteroid titre in the haemolymph. However, the ecdysteroid titre in the KO female pupae was the same as or even higher than that in the wt pupae (Supplementary Fig. S4). Therefore, the observed reduction in the ovary weight of the KO females was not caused by a reduced ecdysteroid signal but rather by a lack of BIGFLP itself, although ecdysteroids may be essential in the initial stage of the induction of ovarian development. Thus, we speculate that the increase in ecdysteroids after pupal ecdysis initiates ovarian development and, at the same time, induces BIGFLP secretion from the fat body, thereby promoting the growth and development of the ovaries.

## Methods

### Animals

A bivoltine strain of *B*. *mori* (Kosetsu) was reared at 25 ± 1.0 °C under a 12-h light and 12-h dark photoperiod and a relative humidity of 50–70% on an artificial diet ‘Silkmate’ (Nihon Nosan Kogyo, Yokohama, Japan). Most larvae (>95%) started wandering on day 5 of the final instar and ecdysed to pupae on day 8. Most pupae (>90%) eclosed at 8 days (males) or 9 days (females) after pupation. During screening, larvae of the KO strains were reared at 25 ± 1.0 °C under a 20-h light and 4-h dark photoperiod to induce non-diapause eggs as described previously^29^.

### Genome editing with the CRISPR-Cas9 system

The Eco-RI-SphI fragment, which contains the U6 promoter and a guide RNA sequence, was subcloned from pBFv-U6.2^30^ into pUC18 and used for sgRNA synthesis. Two oligo-DNAs of each of two target sites of *BIGFLP* #1 (5′-CTTCGGCCACCGTCAGTAGAACG-3′; 5′-AAACCGTTCTACTGACGGTGGCC-3′) and #2 (5′-CTTCGATCCTCCTCGTTCTACTGA-3′; 5′-AAACTCAGTAGAACGAGGAGCATC-3′) were heated at 100 °C for 5 min and left at room temperature for annealing. Each target sequence was then inserted into the Bbs1 restriction site between the U6 promoter region and the guide RNA sequence of the vector. The target sequences were amplified using either the T7-#1 forward primer (5′-CTAATACGACTCACTATAGGCCACCGTCAGTAGAA-3′) or the T7-#2 forward primer (5′-CTAATACGACTCACTATAGATCCTCCTCGTTCTAC-3′) and the guide RNA reverse primer (5′-AAAAAAGCACCGACTCGGTGC-3′). sgRNA was then synthesised using the MEGAscript® RNAi Kit (Thermo Fisher Scientific, MA, USA). Cas9 mRNA was purchased from Thermo Fisher Scientific. sgRNA and Cas9 mRNA were each injected at a concentration of 250 ng/µL (10–20 nL/egg) and mutant strains were analysed after three serial backcrosses to the wt strain. Microinjection and mutant screening were performed according to Shiomi *et al*.^31^.

### Haemolymph collection and hormone detection

The haemolymph was collected from day-0 pupae for BIGFLP detection by Western blotting. Western blotting was carried out as previously described by Okamoto *et al*.^13^, with the exception that the anti-BIGFLP antibody D7H3 was used in place of the anti-bombyxin antibody M7H2 as the primary antibody. The haemolymph was also collected daily at 6 h after lights-on for 8 days after pupation (P0 to P7) and the titres of BIGFLP and ecdysteroids were determined by TR-FIA, as described previously^17,32^.

### Phenotypic analyses

The body weight of each individual was measured daily at the same time of day (2.5 h after lights-on) from day 0 of the fifth instar to day 0 of the adult stage. Images of the male genitalia and adult appendages (antennae, forelegs and forewings) were obtained with a Nikon D5000 (Nikon, Tokyo, Japan) or Finepix S9900W (Fujifilm, Tokyo, Japan) digital camera. The lengths of the male genitalia, antennae and forelegs (tibia and tarsus) and the area of the forewings were then measured from the images. The ovaries and testes were dissected daily from pupae at 4–8 h after lights-on and weighed.

### Egg laying

Adults were mated for 2 h at 25 °C under light conditions. The lights were then switched off for 24 h, during which time the females laid their eggs on egg boards at 25 °C. The numbers and sizes of the laid eggs were measured using images obtained using a Finepix S9900W digital camera.

### BIGFLP injection

BIGFLP was purified from the pupal haemolymph as described previously^17^. From day 0–6 female pupae were anesthetized by submerging them in water for 30 min. BIGFLP was diluted with deionized water containing 0.1% bovine serum albumin, and 0.75 or 1 µg of the hormone (25 µl) were injected into the dorsal intersomitic region of the pupae. Control pupae were injected with the vehicle.

### Detection of phosphorylated Akt

Western blotting was performed to detect phosphorylated Akt, Akt and alpha-tubulin, as described previously^17^. The signal intensities of phosphorylated Akt and Akt were measured using Image Lab™ version 4.1 software (Bio-Rad, Hercules, CA, USA).

### Ovary transplantation and brain removal

KO (#1–8) and wt females were water-anesthetized for 30 min within 6 h of pupation. For ovary transplantation, one ovary was removed from each individual. A wt or #1–8 ovary was then implanted in different individuals. For brain removal, the tip of the head was cut open, and the brain was removed. The wound was sealed with paraffin. After eclosion, the implanted ovaries were dissected, photographed using a Finepix S9900W digital camera and weighted.

### Statistical analyses

All data are presented as means ± standard deviations (SD). Student’s *t*-test was used for the comparison of two groups and Dunnett’s or Tukey–Kramer’s tests were used for multiple comparisons.

## Supplementary information

Supplementary information

## Data Availability

The datasets in the current study are available from the corresponding author on reasonable request.
