# Supplementary material for: An insulin-like growth factor-like peptide promotes ovarian development in the silkmoth Bombyx mori
Source: Sci Rep. 2019 Dec 5;9:18446. doi: 10.1038/s41598-019-54962-w (PMC6895095; doi:10.1038/s41598-019-54962-w)
Supplement: Supplementary file 1 — Supplementary information [file 41598_2019_54962_MOESM1_ESM.pdf]

(Supplementary information)

An insulin-like growth factor-like peptide promotes ovarian development in the silkmoth *Bombyx mori*

Daiki Fujinaga[1], Kunihiro Shiomi[2], Yoshimasa Yagi[3], Hiroshi Kataoka[1 *] & Akira Mizoguchi[4 *]

[1]Department of Integrated Biosciences, Graduate School of Frontier Sciences, The University of Tokyo, Kashiwa, Chiba 277-8562, Japan. [2]Faculty of Textile Science and Technology, Shinshu University, Ueda 386-8567, Japan. [3]Division of Biological Science, Graduate School of Science, Nagoya University, Nagoya 464-8602, Japan. [4]Division of Liberal Arts and Sciences, Aichi Gakuin University, Nisshin, Aichi 470-0195, Japan. Correspondence and requests for materials should be addressed to H. K. (email: kataoka@edu.k.u-tokyo.ac.jp) or A. M. (email: amizo@dpc.agu.ac.jp)

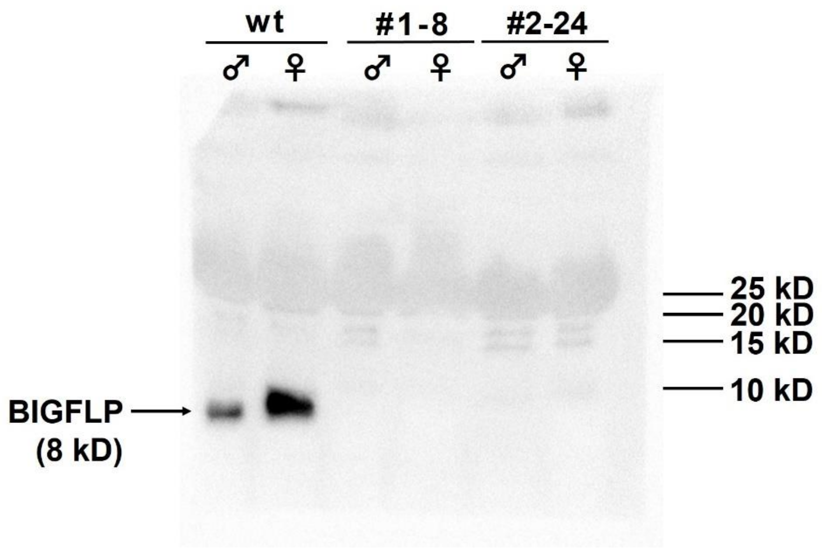

Supplementary Figure S1. The Original scan for the blots in Figure 1c.

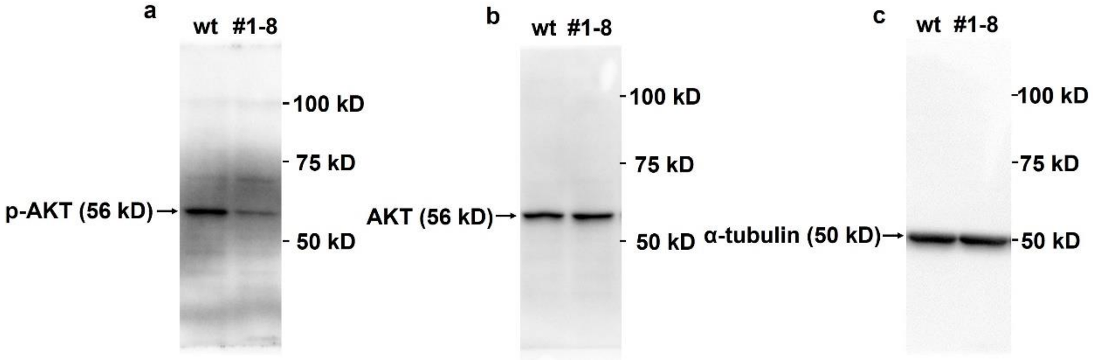

Supplementary Figure S2. Original scans for the blots in Figure 4a. The original blots to detect phosphorylated AKT (p-AKT) (**a**), AKT (**b**) and α-tubulin (**c**) of Figure 4a are shown.

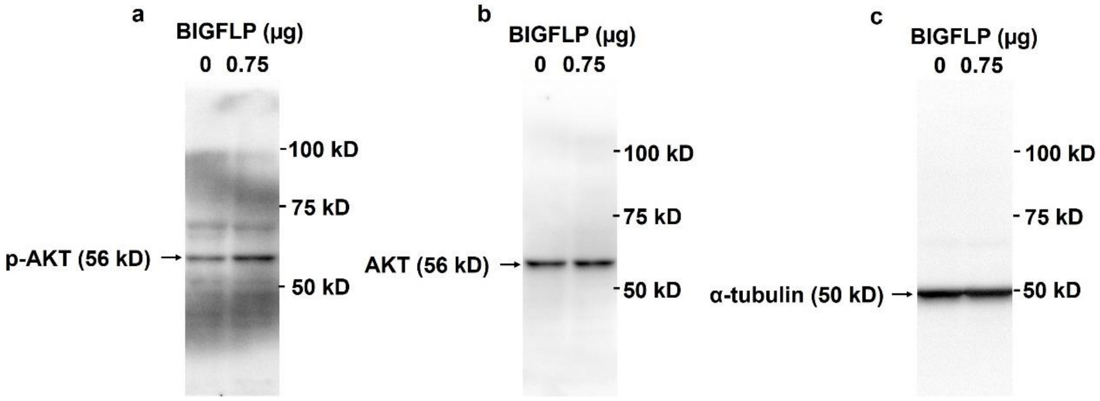

Supplementary Figure S3. Original scans for the blots in Figure 4c. The original blots to detect phosphorylated AKT (p-AKT) (**a**), AKT (**b**) and α-tubulin (**c**) of Figure 4c are shown.

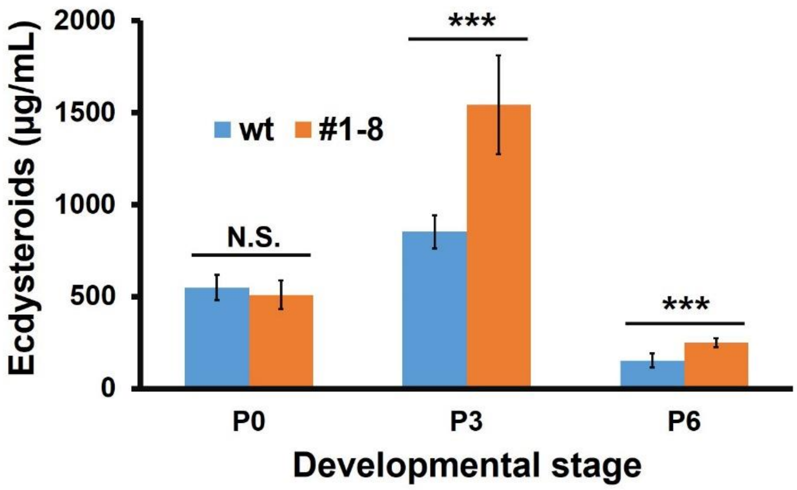

Supplementary Figure S4. Comparison of the ecdysteroid titres in the haemolymph of wt and KO *B. mori* females. The titres were determined by TR-FIA and are expressed as 20E equivalents. Values are means ± SD (n = 4). Asterisks indicate significant differences between wt and #1–8 (Student's *t*-test: ***, $p < 0.001$; N. S., not significant).
